# The CC′ loop of IgV domains of the immune checkpoint receptors, plays a key role in receptor:ligand affinity modulation

**DOI:** 10.1038/s41598-019-54623-y

**Published:** 2019-12-16

**Authors:** Shankar V. Kundapura, Udupi A. Ramagopal

**Affiliations:** 1 Division of Biological Sciences, Poornaprajna Institute of Scientific Research, #4, 16th Cross, Sadashivnagar, Bangalore 560080 India; 2 Manipal Academy of Higher Education, Manipal, Karnataka 576104 India

**Keywords:** Protein design, X-ray crystallography

## Abstract

Antibodies targeting negative regulators of immune checkpoints have shown unprecedented and durable response against variety of malignancies. While the concept of blocking the negative regulators of the immune checkpoints using mAbs appears to be an outstanding approach, their limited effect and several drawbacks, calls for the rational design of next generation of therapeutics. Soluble isoforms of the negative regulators of immune checkpoint pathways are expressed naturally and regulate immune responses. This suggests, affinity-modified versions of these self-molecules could be effective lead molecules for immunotherapy. To obtain better insights on the hotspot regions for modification, we have analysed structures of 18 immune receptor:ligand complexes containing the IgV domain. Interestingly, this analysis reveals that the CC′ loop of IgV domain, a loop which is distinct from CDRs of antibodies, plays a pivotal role in affinity modulation, which was previously not highlighted. It is noteworthy that a ~5-residue long CC′ loop in a ~120 residue protein makes significant number of hydrophobic and polar interactions with its cognate ligand. The post-interaction movement of CC′ loop to accommodate the incoming ligands, seems to provide additional affinity to the interactions. *In silico* replacement of the CC′ loop of TIGIT with that of Nectin-2 and PVR followed by protein docking trials suggests a key role of the CC′ loop in affinity modulation in the TIGIT/Nectin pathway. The CC′ loop appears to be a hotspot for the affinity modification without affecting the specificity to their cognate receptors.

## Introduction

The concept of blocking the negative regulators of the immune system (checkpoint blockade) to control/eradicate cancer and its durable response over the plethora of cancer types, has revolutionised the way cancer is treated today. Here, the reactivation of the immune system to fight cancer lies at the core of its philosophy. The involvement of co-stimulatory or inhibitory immune checkpoint receptors (ICRs)^1–3^, along with primary MHC-2 (Major Histocompatibility Complex-2) and TCR (T-cell receptor) signalling, is paramount for the activation or deactivation of T-cells. These immune checkpoint pathways like CTLA-4/CD28:B7-1/B7-2, CD226/TIGIT:Nectin-2/PVR and PD-1:PD-L1/PD-L2, hold considerable sway over the functional fate of the T-cells, B-cells, NK cells and so on^4^. Interestingly, tumour cells have been known to exploit some of these pathways by over-expressing the ligands of the inhibitory ICRs to escape immunosurviellance^5^. The blockade of signalling of these negative pathways through antibodies resulted in the reinvigoration of exhausted T-cells^6^, which eventually led to the regression of tumour size^7–9^.

Extraordinary amount of research on this conceptually novel approach and subsequent preclinical and clinical studies have resulted in the approval of effective monoclonal antibodies (mAbs) against inhibitory ICRs, as cancers therapeutics^10–14^, which was pioneered by the first-in-class mAb called Yervoy^15,16^. Immune checkpoint blockade via monoclonal antibodies became so influential in treating cancer that the Nobel prize for Physiology/Medicine 2018 was awarded to James P. Allison and Tasuku Honjo ‘*for their discovery of cancer therapy by inhibition of negative immune regulation*’. As the popularity of mAbs in cancer therapy grew, so did the reports of their drawbacks namely, high cost^17^, poor solid tumor infiltration of these mAbs^18^, moderate to severe adverse effects^19^ and the observation that the mAb based therapy is responsive only in minority of patients^20^. Further, recent findings of resistance to mAbs mediated immunotherapy is worrisome^21^. The adverse effects observed from the clinical trials of TGN1412, an antiCD28 mAb^22,23^ highlight the unpredictable consequences of such antibodies. Moreover, these antibodies have a long half-life and are administered in high dose. Hence, in the event of adverse effect of these antibodies, it is difficult to quickly withdraw them from the body^24^. Further, the gaps in understanding of the underlying biological mechanisms is another major setback for its logical application. For example, the observation that, distinct cellular mechanisms play a role in anti-CTLA-4 and anti-PD-1 checkpoint blockade recommends further investigations on fundamental mechanisms^25^. While the concept is undoubtedly a breakthrough, the above limitations suggests that we are yet to experience the full potential of immune checkpoint blockade which calls for the urgent exploration of novel modalities that can target the same pathways. There has been an encouraging shift from the use of mAbs to small molecules^26^, DNA aptamers^27^, peptides^28^ and modified ICRs^18,29,30^, some of which are in the preliminary stages of investigation and have immense potential to be on the shelf of a pharmacy in the near future.

Use of self-proteins for therapeutic purposes started with insulin and today, several such proteins are being used for various clinical applications^31^. Further, the soluble versions of immune checkpoint receptors (ICRs) as Fc conjugates is a proven non-mAb based approach, which is already in clinical practice. To name a few, Orencia (Abatacept-soluble CTLA-4-IgG1Fc), Nulojix (Belatacept, a more efficacious version of Orencia) and Etanercept (commercial name Enbrel-soluble TNF receptor-IgG1Fc) are approved for treatment of moderate to severe rheumatoid arthritis^32^. The success of Etanercept in treating the autoimmune disorder was widely acknowledged, so much so that it became the 3^rd^ largest selling drug in 2015^33^. In fact, soluble isoforms of ICRs are secreted by the immune cells to regulate immune responses^34,35^. This suggests that these isoforms which are naturally present in our body, could be rationally modified to engineer lead molecules which function similarly to mAbs. There have been interesting reports of modified ICRs which have increased binding affinity and penetrance in solid tumours when compared to mAbs. For instance, a high affinity consensus (HAC) Programmed cell death protein-1 (PD-1) mutant displayed 35,000-fold higher binding affinity to one of its ligands, PD-L1 (Programmed cell death protein-1 ligand 1) than the wild type PD-1^18^. The HAC PD-1 is reported to have better penetrance into solid tumours than the anti-PD1 mAb and displayed its potential to be a diagnostic tool for PD-L1 expressing tumours. However, this 10-point mutant was obtained from a tedious directed evolution method, which had lost its binding affinity to its other ligand PD-L2 (Programmed cell death protein-1 ligand 2). Contrary to this example, a structure based rational design of single point PD-1 mutant (A_132_L) displayed a 45-fold and 30-fold increased binding affinity to both its ligands, PD-L1 and PD-L2 respectively^29^. Hence, it appears that the structure-based rational design provides an opportunity to create lead molecules with tuneable affinity without affecting the specificity to their cognate partner(s). However, for such an endeavour, a sound structural knowledge of the immune receptor:ligand complexes, is paramount.

In most of the ICRs, the membrane distal ectodomain involved in immune regulation are derived from a similar scaffold having IgV (Immunoglobulin V) domain fold. For example, in co-inhibitory molecules such as PD1, PD-L1, PD-L2, CTLA-4, CD28, B7-1, B7-2, TIM3, LAG3, TIGIT, PVR and so on, the membrane distal interacting domains are carved from an IgV domain. The IgV domain belongs to a functionally variant large superfamily called the immunoglobulin superfamily (IgSF: Please refer to Supplementary Information for more information on architecture of Ig domains and their classifications).

This study aims at understanding the hotspot regions of receptor:ligand interaction to derive important insights that can be incorporated in the design of therapeutic ICRs to achieve optimal binding affinity without affecting specificity. Our goal is not directed towards the design of molecules with too many modification and exorbitant affinity to their cognate partners, as there is a chance of undesirable cross-interactions with other IgV domain proteins. Instead, optimal increase in binding affinity towards the targeted ligand without sacrificing the specificity is desirable^36^. A small, yet significant increase in binding affinity is shown to increase the biological potency of the molecule by many folds^37^. Towards this goal, we have analysed the available structures of immune receptor:ligand complexes containing IgV domain(s). It was interesting to observe that CC′ loop, which is not one of the key loops (CDRs) in IgV domain of antibodies, appears to be an active determinant of receptor:ligand interaction in the case of ICRs, which is further supported by existing mutagenesis studies on CC′ loop. To our surprise, the importance of the CC′ loop in ligand binding has been sparsely highlighted when compared to FG loop and the front face. To further authenticate our observation, TIGIT:Nectin-2/PVR pathway was considered to elucidate the importance of CC′ loop as TIGIT binds to itself and its partners in the same orientation and yet has differing binding affinities^38^, making this pathway attractive to computationally investigate the effect of modification of the CC′ loop on binding affinity. The CC′ loop of TIGIT was spliced with the CC′ loops of Nectin-2 and PVR, to investigate its effect on the binding affinity with PVR, Nectin-2 and TIGIT (homodimer).

Here, by employing structural bioinformatics tools, we found a sizeable number of IgV domain containing ICRs which use their CC′ loops to make indispensable interactions with their cognate ligands. Overall, this study attempts to shed light on the importance of CC′ loop in few of the important IgV/Ig like domain containing ICR pathways and provide insights on effective rational modification of self-molecules for immunotherapy.

## Results and Discussion

Literature and structural search led to the identification of 18 ICR complexes which contained at least one IgV domain. Of the 18 ICR complexes, 33 IgV domains, 2 small molecules (phosphatidylserine and siallylactose) and 1 TNF superfamily member (HVEM of BTLA-HVEM complex) were present. Among them, 33 IgV domains were considered for further analysis on their mode of interaction with their cognate partners. In this analysis, the parameters used to ascertain the contribution of CC′ loop and FG loop to the interaction-interface are buried surface area (BSA) and inter-protein hydrophilic interactions. Further, post-interaction movement of CC′ loop and FG loop (C_α_ atom movement >3 Å) upon binding, was taken into consideration.

### The association of IgV domains in antibodies and ICR complexes

**Figure 1:**
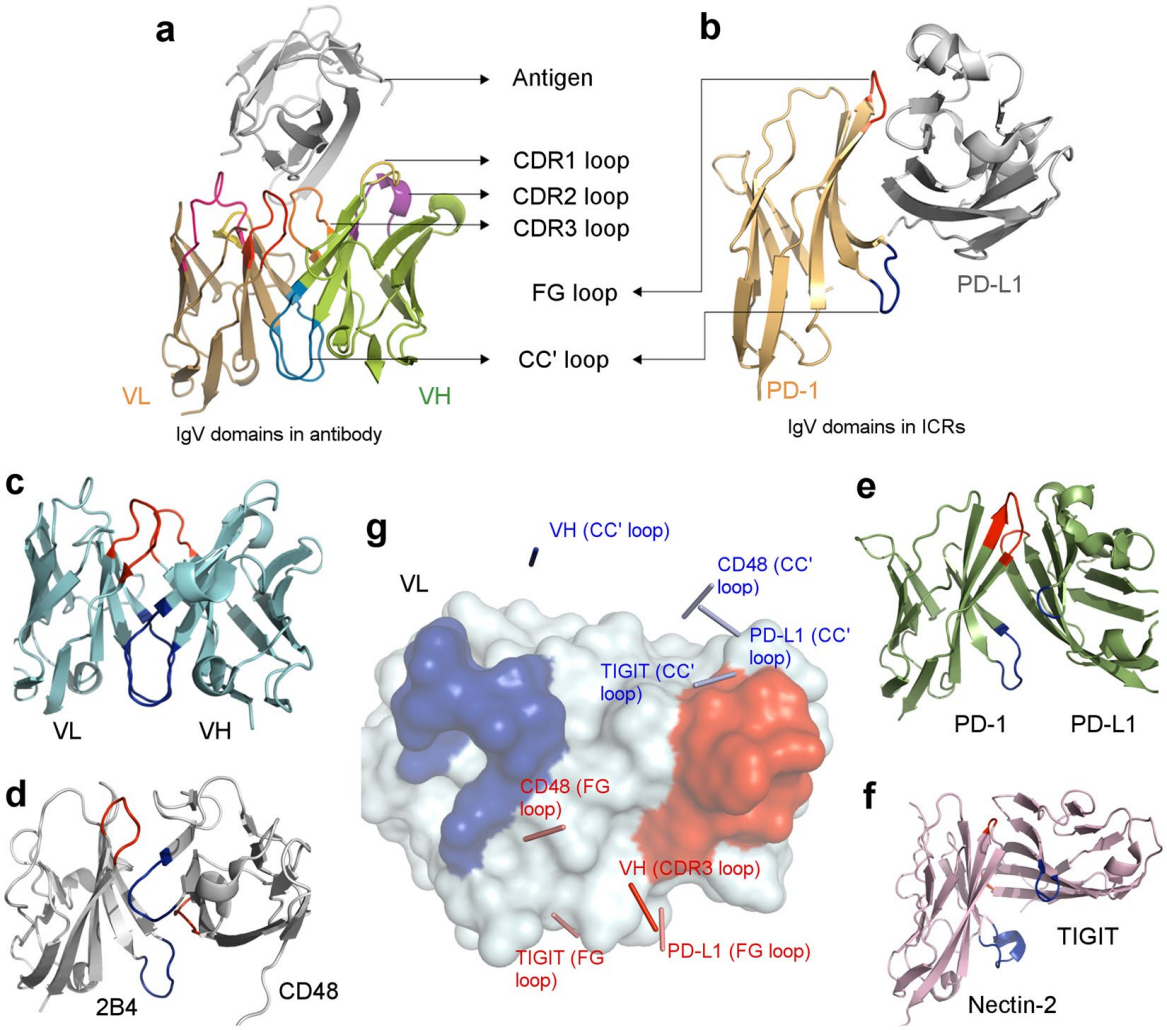
Association of IgV domains in antibodies and ICR complexes: The arrangement of the CDR loops and CC′ loops of the V_H_ and V_L_ domains of antibodies is shown in panel a, where only CDR loops interact with the antigen. Panel b illustrates the positions of CC′ loop and FG loop of the IgV domain of ICR (PD-1:PD-L1) where the FG loop, front face and CC′ loop make key interactions with its cognate ligand. Panel c illustrates V_H_ and V_L_ domains of an antibody presented in cartoon representation (teal), where CC′ loop is coloured dark blue (in all panels of Fig. 1) and FG loop, red (in all panels of Fig. 1). Panel d illustrates the 2B4:CD48 complex (grey) where positions of CC′ loops (dark blue) and FG loop (red) are marked. The same is illustrated in PD-1:PD-L1 (forest green) shown in panel e and TIGIT:Nectin-2 (deep salmon) shown in panel f. The V_L_ domain (surface, teal, panel g) of the antibody was superposed with PD-1 of PD-1:PD-L1 complex, Nectin-2 of the TIGIT:Nectin-2 complex, 2B4 of the 2B4:CD48 complex. The position of CC′ loops and FG loops of V_H_ domain, PD-L1, TIGIT and CD48 are marked by ribbon representation, shown as thin rods connecting two central residues of the respective loops (Panel g).

In antibodies, each Fab (fragment of antigen binding) is a heterodimeric association of two IgV domains (termed as V_H_ and V_L_) followed by association of IgC domains of light and heavy chains. As shown in the Fig. 1a, the two IgV domains associate through their hydrophobic front face, where the CC′ loops (Fig. 1a; shown in blue) from the V_H_ and V_L_ domains interact with each other and are a part of extended hydrophobic core. Further, the CC′ loops are distal to the bound antigen; whereas the FG loops [or CDR3 of V_H_ (orange) and V_L_ (red)] located at the other end of the front face (Fig. 1a) make key interaction with the antigen^39^.

This mode of interaction of V_H_ and V_L_ domains of a Fab brings their respective variable loops (CDR1, CDR2, CDR3) in proximity to each other. These closely placed CDR loops and their variation endows ability to recognize diverse antigens with high specificity (Fig. 1a)^40–43^.

However, in large number of ICRs and other receptors, these IgV domains are the N-terminal domain which interacts with their cognate partner. Consequently, the front face is relatively polar in IgV domains of ICRs, and predominantly utilizes the membrane distal FG loop (corresponding to CDR3 loop in antibodies), the flat front-face comprising of G, F, C, C′ and C′′ (and A′ strand in most cases) strands and the membrane proximal CC′ loop for binding to their respective binding partner (Fig. 1b), unlike the IgV domains of antibodies which predominantly use the CDR loops to recognize an antigen.

**Figure 2:**
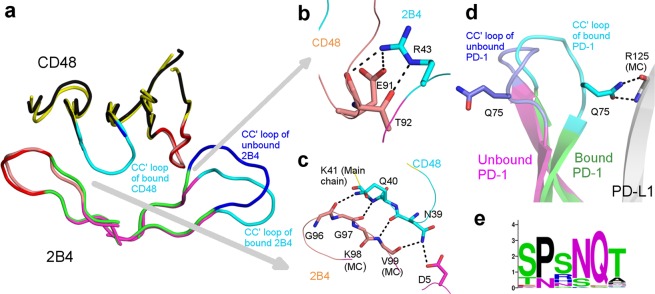
Significant post-interaction movement in CC′ loop: Panel a illustrates the superposition of 2B4 (ribbon, black):CD48 (ribbon, magenta) complex with apo structures of 2B4 (ribbon, yellow) and CD48 (ribbon, green), where CC′ loops of bound 2B4 and CD48 is shown in cyan and unbound 2B4 is shown in dark blue (only relevant part is shown for clarity). The CC′ loop of apo CD48 is not represented as it is not modelled in the PDB structure (PDB:2PTV). The FG loops of bound 2B4 and CD48 is shown in salmon and that of the apo structures is shown in red. Panel b illustrates the interactions of R_43_ (stick, cyan) of the CC′ loop of 2B4 with CD48 (stick, salmon). Panel c illustrates the interactions of the CC′ loop of CD48 (stick, cyan) with 2B4 (stick, salmon). Panel d illustrates the post-interaction movement of the CC′ loop (cyan) of PD-1 (cartoon, green) upon interaction with PD-L1 (cartoon, grey) when compared with the CC′ loop (dark blue) of apo PD-1 (cartoon, magenta). The sequence logo of residues of the CC′ loop of PD-1 is depicted in panel e.

Although, the association of two IgV domains (V_H_ and V_L_) in antibodies and those seen in ICRs complexes are mediated by the front face and the mode of their interaction is considerably similar, our analysis suggests that several aspects of their association are quite different from each other. In antibodies, the CC′ loop from two associating IgV domains are always close to each other and are involved in the formation of heterodimeric hydrophobic core, which restricts relative orientation of two IgV domains. Interestingly, no such unique orientation is observed in the interactions of IgV domains of different ICR complexes. In fact, almost every ICR complex has a different binding orientation where the positions of CC′ loops and FG loops are variable (Fig. 1c–g). This is illustrated well in the case of m2B4-mCD48 complex (PDB ID: 2PTT) where the CC′ loop of mCD48 is found near the vicinity of the FG loop of m2B4 and vice versa (Fig. 2d,g). In some cases, for example homodimeric NTB-A, two CC′ loops are close to each other, whereas the FG loops are far away^44^. These different binding orientations observed in ICRs point towards the varying degree to which the CC′ loop is involved in the interaction interface. In ICR complexes, the role of the front face, FG loop and the CC′ loop are much like the role of palm (front face), index-finger (FG loop) and thumb (CC′ loop) of a clapping hand. Hence, the FG loop and the CC′ loop flanking the front face act as clamps in order to hold the incoming binding partner. This analysis, together with existing mutagenesis studies (Supplementary Table 1), suggests that the CC′ loop makes vital interactions that fine-tune the affinity between the interacting IgV domains, whereas the FG loop and the front face provide the required specificity.

Based on these observations, we analysed 18 ICR complexes and it was observed that the CC′ loop of about 49% of the proteins analysed, contributed more than 10% to the total BSA of interaction interface of one IgV domain. This is an important statistic as the average length of the CC′ loop is only 5 residues long. Hence, it is interesting to observe how a ~5-residue long loop in a ~120 residue protein contributes more than 10% of the total BSA of the interaction interface in nearly half of the ICR complexes analysed. Additionally, along with the key BSA contributions, about 27% of the 33 proteins analysed, had hydrophilic interactions like hydrogen bonds and salt bridges contributed by the CC′ loop towards the interaction interface. It was also observed that about 24% of the proteins considered for this study, had more BSA contribution from CC′ loop than the FG loop towards the interaction interface (Table 1). These statistics hints at the considerable influence of CC′ loop in the interaction-interface of these complexes.

**Table 1:** Interaction analysis of 18 IgV domain containing ICR complexes.

Complex	PDB ID	Monomer	Length of CC′ loop	H-Bond interactions of CC′ loop	BSA of interacting residues of CC′ loop (Å^2^)	Interface BSA of monomer (Å^2^)	% BSA contribution of CC′ loop	% BSA contribution of FG loop	Post-interaction movt. of CC′ loop#	Post-interaction movt. of FG loop
TIGIT dimer	3RQ3	TIGIT (A)	4	0	0	659.7	0	10.06	No	No
		TIGIT (B)	2	0	0	670.3	0	10.28		
Nectin-2 dimer	4HZA	Nectin-2 (A)	9	0	34.23	914.9	3.74	8.70	No	No
		Nectin-2 (B)	9	0	30.33	928.4	3.32	8.27		
TIGIT:Nectin-2	5V52	TIGIT	2	0	0	794.6	0.00	9.48	No	No
		Nectin-2	9	0	21.14	785.5	2.66	7.53	Yes, 3.28 Å C_α_ N78	
TIGIT:PVR	3UDW	TIGIT	2	0	0	799.3	0.00	7.21	No	No
		PVR	7	3	123.4	816.6	15.44*	8.28	Yes, 3.59 Å C_α_ E71	
NTB-A dimer	2IF7	NTB-A (A)	2	1	62.33	751.6	8.29*	6.72	ND	ND
		NTB-A (B)	2	2	59.06	750.3	7.87*	5.30		
CD84 dimer	2PKD	CD84 1	3	0	38.36	738.8	5.19	8.68	ND	ND
		CD84 2	3	0	38.31	736.6	5.20	8.36		
CD2:CD58	1QA9	CD2	7	2 H, 2SB	169.96	698.3	24.34*	22.82	No	No
		CD58	2	1 H, 2SB	76.56	657.5	*11.64*	15.94	No	No
2B4:CD48	2PTT	2B4	6	2 H, 1SB	139.79	735.5	*19.01*	21.35	Yes, 4.2 Å C_α_ H42	No
		CD48	5	5	206.72	714.9	*28.92*	30.46	Disordered to ordered	No
CRTAM dimer	3RBG	CRTAM (A)	5	0	82.97	729.2	11.38*	8.82	ND	ND
		CRTAM (B)	5	0	82.34	713.8	11.54*	10.77		
CRTAM-Necl-2	4H5S	CRTAM	5	2	136.88	771.2	17.75*	6.57	No	No
		Necl-2	5	1	100.1	745.8	*13.42*	29.30	ND	ND
B71:CTLA-4	1I8L	B71	2	1	78.87	590.5	*13.36*	13.94	No, but K36 stabilizes	No
		CTLA-4	6	0	0	644.5	0.00	44.00	No	
B72:CTLA-4	1I85	B72	5	1	61.5	566.9	*10.85*	44.62	No	Yes, 3.40 Å C_α_ T93
		CTLA-4	10	0	0	666.2	0.00	79.38	No	No
hPD-1:hPD-L1	4ZQK	hPD-1	5	4	121.08	795.5	*15.22*	17.41	Yes, 8.23 Å C_α_ Q75	No
		hPD-L1	2	0	0	752.3	0.00	3.27	No	No
mPD-1:mPD-L2	3RNK	mPD-1	5	3	133.95	980.6	*13.66*	31.01	Yes, 3.2 Å C_α_ S41	Yes, 5.62 Å C_α_ P97
		mPD-L2	11	0	71.35	966.5	7.38	9.36	Yes, 9.5 Å C_α_ D65	No
mPD-1:hPD-L1	3SBW	mPD-1	5	5	148.63	873.5	*17.02*	25.03	No	No
		hPD-L1	2	0	0	852.1	0.00	1.36	Yes, 3.18 Å C_α_ 61D	Yes, 4.32 Å C_α_ G119
mTIM-3	3KAA	mTIM-3	11	2	61.29	195	*31.43*	57.13	Yes, 6.6 Å C_ζ2_ W41	No
CD22	5VKM	CD22	4	0	10.44	276.8	3.77	13.81	No	No
BTLA-HVEM	2AW2	BTLA	4	1	109.53	940.4	11.65*	3.48	ND	ND

Entries in italics indicate 10% or higher BSA contribution of the CC′ loop to the interaction-interface. Entries with * denote BSA contribution of CC′ loop higher than that of FG loop. Note that the residues that are labelled as loop in PDB header file and which connects the C and C′ strands are considered as CC′ loop. The same was considered for FG loop. ^#^Post-interaction movement of any C_α_ atoms of CC′ loop and FG loop of more that 3 Å is considered as significant. Movement is abbreviated as “Movt.” and not determinable is abbreviated as “ND”.

Post-interaction conformational changes or the induced fit of CC′ loop was another interesting feature that was observed. About 27% of the analysed ICRs showed deviation of more than 3 Å of the C_α_ atom of at least one of the residues in the CC′ loop before and after interaction. This value is only 9% in the case of FG loop. Further, the extent of post-interaction movement of CC′ loop is significantly larger when compared to that of the FG loop where, maximum deviation of 9.5 Å and 5.6 Å have been observed for CC′ loop and FG loop respectively. This difference seems to indicate that the CC′ loop is subjected to more post-interaction movements to accommodate the incoming ligand than FG loop. It must be pointed out that alongside front face, the FG loop appears to play a pivotal role in providing the specificity of the ICR interactions. This is observed in TIGIT:TIGIT/Nectin-2/PVR pathway where a conserved _112_TYP_114_ motif present on the FG loop of TIGIT helps in the lock-and-key interaction with its ligands Nectin-2^38,45^, Polio virus receptor (PVR) and itself^46^. Similarly, the FG loop of hCTLA-4 and hCD28 with a rigid _99_MYPPPY_104_ motif provides specificity to their cognate receptors, B7-1 and B7-2^47^. Hence, it could be hypothesized while FG loop is involved in conferring specificity, CC′ loop’s ability to undergo ligand induced conformational changes might be contributing to additional affinity.

As depicted in the Table 1, it is evident that the CC′ loop appears to contribute to ligand:receptor interactions in ICR complexes and in some cases, ligand induced conformational changes appear to be an important factor. In the case of PVR, it has also been shown that its ligands TIGIT and CD96^48^ induce distinct CC′ loop movement upon complexation. The same CC′ loop is disordered in one of the two PVR:DNAM-1 complexes observed in the asymmetric unit^49^, suggesting the versatility of CC′ loop and its possible contribution to varied receptor:ligand binding affinities. Hence, based on the role of CC′ loop and intricacies of the post-interaction conformation changes in the CC′ loop, some interesting features were highlighted, 1) Significant post-interaction movement of CC′ loop 2) Loop to β-strand transition of CC′ loop upon interaction 3) Unusual conformation of CC′ loop leading to non-canonical IgV domain. In the following sections, we provide few examples of post-interaction conformational changes and two examples of unique variation in CC′ loop leading to non-canonical conformation that carve a binding pocket for small molecules.

### Significant post-interaction movement in CC′ loop

In murine 2B4:CD48 complex (Fig. 2a), 2B4 is a member of signalling lymphocyte activation molecule (SLAM), and a receptor expressed on NK cells. SLAM family members are considered as important targets for anti-myeloma immunotherapy^50^. 2B4 positively regulates NK cell function and makes heterophilic interaction with CD48^51^. This complex is involved in Interleukin-2 associated propagation and activation of NK cells which leads to NK cell mediated cytotoxicity and tumour clearance^52^. The CC′ loop of the m2B4 comprising of residues _38_EQGSHR_43_ (PDB:2PTT)^51^ contributes 19.01% to the total 2B4 interaction-interface BSA. This CC′ loop is also seen to contribute two potential hydrogen bonds and one salt bridge (Fig. 2b). Furthermore, the CC′ loop of CD48 comprising of _37_TKNQK_41_ (PDB:2PTT) contributes 28.92% to the total CD48 interaction-interface BSA, along with five potential hydrogen bonds (Fig. 2c). It appears that the observation of post-interaction movement of CC′ loop of 2B4 and CD48 is unique in this context (Fig. 2a). The CC′ loop of 2B4 is reported to be flexible in its apo form, among the four molecules in the asymmetric unit, this loop is not modelled in two molecules due to its disordered state. However, in the complex, the CC′ loop of 2B4 moves to accommodate CD48 and interacts with the 2B4, as mentioned earlier. This movement is characterized by the maximum deviation of about 4.2 Å of C_α_ atom of H_42_, when apo (PDB:2PTU) and bound 2B4 (PDB:2PTT) were compared (Fig. 2a). As observed in the 2.4 Å structure, the CC′ loop of apo CD48 is highly flexible and was not modelled in any of the molecules in its asymmetric unit. However, upon complexation (as observed in 1.6 Å structure), the CC′ loop assumes a rigid hook like conformation and latches on to 2B4 (Fig. 2c). This is a unique feature where CC′ loops of both 2B4 and CD48 show post-interaction movements unlike FG loops of these proteins, which contribute 28.51% (2B4) and 30.89% (CD48) to the total interaction-interface BSA. Since, the CC′ loops of 2B4 and CD48 are located on opposite sides of each other, the post-interaction movement of both the CC′ loops seem to act like clamps to increase the binding affinity of the 2B4:CD48 interaction.

A similar post-interaction movement of the CC′ loop is seen in human PD-1 (hPD-1) when it interacts with human PD-L1 (hPD-L1)^53^. PD-1 is a well-studied inhibitory cell surface receptor^54^ inducibly expressed on T-cells, NK cells, B-cells, dendritic cells and macrophages^55–57^. Its ligands PD-L1 and PD-L2 have been observed to be over-expressed on several kinds of tumour cells to escape immunosurveillance^58–60^. The CC′ loop of hPD-1 comprising residues _71_SPSNQ_75_ (PDB:4ZQK) contributes 15.22% to the total PD-1 interaction-interface BSA and makes four hydrogen bonds with PD-L1. Q_75_ of PD-1 is observed to contribute a BSA of 98.29Å^2^ to the interaction-interface and makes two potential side-chain to main-chain hydrogen bonds with R_125_ of PD-L1 and one side-chain to side-chain hydrogen bond with D_26_ of PD-L1. Interestingly, a large conformational change is observed in the CC′ loop of PD-1 where a maximum deviation of 8.23 Å in C_α_ atom of Q_75_ is observed before and after its interaction with PD-L1 (Fig. 2d). In contrast, there is no post-interaction movement of the FG loop of PD-1 which contributes 17.41% of the total PD-1 interaction-interface BSA. Likewise, no post-interaction movement is seen in the CC′ loop and FG loop of its interacting partner PD-L1. Unlike 2B4:CD48, there is only one-sided clamping of the CC′ loop of PD-1. It is also interesting to note that, of all the residues comprising the CC′ loop, only Q_75_ is completely conserved across most species (Fig. 2e). This opens the possibility to modify other residues of CC′ loop to enhance the binding affinity.

### Loop to β-strand transition of CC′ loop upon interaction

**Figure 3:**
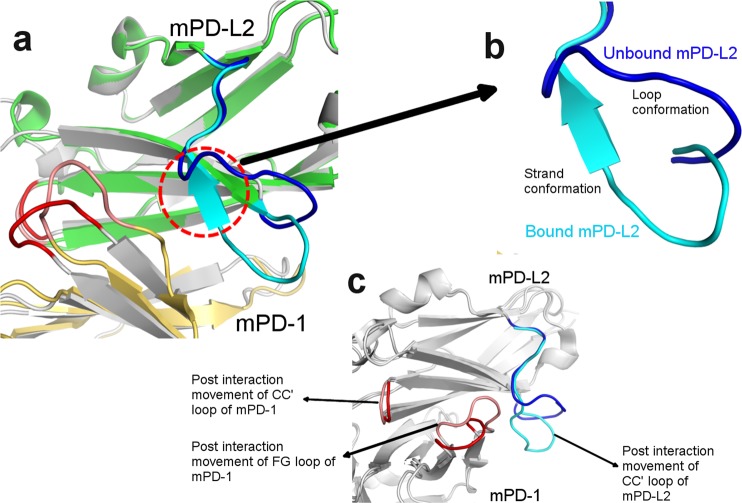
Loop to β-strand transition of CC′ loop: Panel a illustrates the superposition of mPD-1 (cartoon, gold):mPD-L2 (cartoon, green) complex with apo structures of mPD-1 (light grey) and mPD-L2 (grey), only the relevant part is shown for clarity. The CC′ loops of bound and unbound mPD-L2 is cyan and dark blue respectively and the colouring scheme of FG loops are maintained in the same manner as Fig. 2. Panel b depicts the loop to β strand transition of CC′ loop of mPD-L2 upon binding to mPD-1. Panel c highlights the post-interaction movement of the CC′ loop of mPD-L2 and FG loops of mPD-1 and mPD-L2 which causes a three-way clamping.

PD-L2 is another ligand which binds to PD-1 with three times higher affinity than PD-L1^61^. A unique feature in mPD-L2 is observed where, the part of CC′ loop of mPD-L2 undergoes a structural transition from a loop into a β strand conformation, upon binding with its partner mPD-1 (Fig. 3a). The CC′ loop of apo mPD-L2 comprises of residues _62_VENDTSLQSERA_73_ (PDB: 3BOV)^62^, however upon binding to mPD-1, _67_SLQ_69_ of the CC′ loop assumes a β strand conformation (PDB:3BP5)^62^ (Fig. 3b). This could be attributed to the stabilization of the long CC′ loop of mPD-L2 after binding to mPD-1. The conformational change observed in the CC′ loop of PD-L2 is accompanied by a large movement of the CC′ loop of mPD-L2 towards mPD-1, upon binding to mPD-1, which is represented by a deviation of 9.5 Å of C_α_ atom of D_65_ of the CC′ loop of mPD-L2. However, for such a large post-interaction movement, the contribution of the CC′ loop to the total PD-L2 interaction-interface is quite low (7.38%) and we did not observe any polar interactions. A similar feature of post-interaction movements of CC′ loop and FG loop is observed in mPD-1, where a deviation of 3.2 Å in the C_α_ atom of S_41_ and a deviation of 5.62 Å in the C_α_ atom of P_97_, was observed in CC′ loop and FG loop respectively (PDB:3BP5). The CC′ loop and FG loop of mPD-1 contributes 13.66% and 31.01% towards the interaction-interface BSA of mPD-1. The post-interaction movements of CC′ loops of mPD-1 and mPD-L2 and FG loop of mPD-1 causes a three-way clamping which might contribute to the increased binding affinity (Fig. 3c).

### Unusual conformation of CC′ loop leads to non-canonical IgV domain

**Figure 4:**
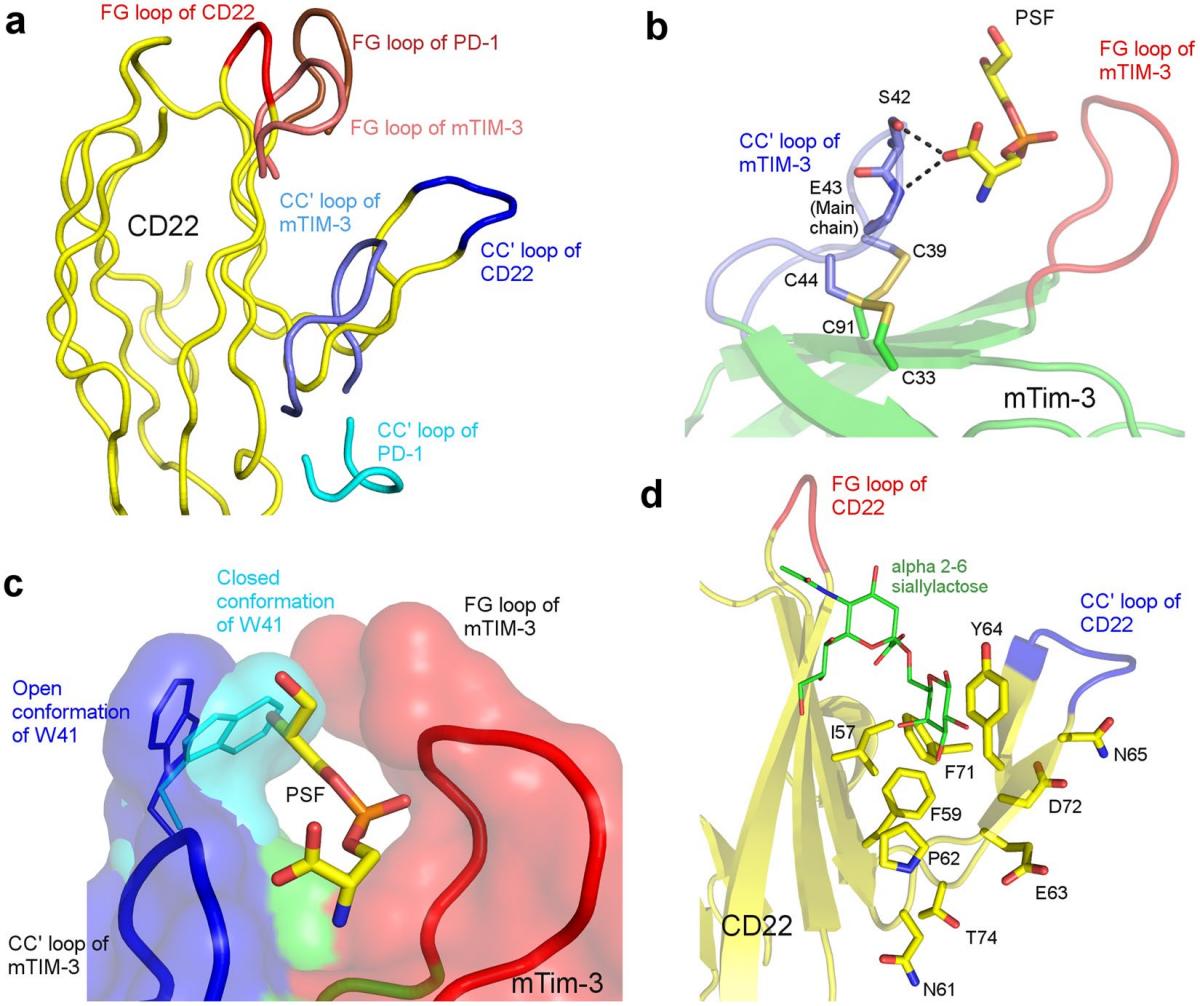
Unusual conformation of CC′ loop leads to non-canonical IgV domain: Panel a illustrates the peeling of CC′ loop (ribbon, dark blue) of CD22 (ribbon, yellow) and CC′ loop (ribbon, Slate) of mTIM-3 in comparison with the canonical CC′ loop of hPD-1 (ribbon, cyan), from the core to form a cleft between FG loop and CC′ loop. This non-canonical conformation of CC′ loops of CD22 and mTIM-3 creates a cleft between the CC′ and FG loops (CD22 (ribbon, red) and mTIM-3 (ribbon, salmon)). Panel b illustrates the non-canonical conformation of CC′ loop (slate) which is stabilised by two non-canonical disulphide linkages (shown in sticks) in mTIM-3. Panel c illustrates the gating of the above-mentioned pocket in mTIM-3 by W_41_, where W_41_ on the CC′ loop assumes a closed conformation (stick and surface, cyan) and is present close to FG loop. W_41_ is flung away from FG loop to assume an open conformation (stick and surface, dark blue) to allow the entry of PtdSer. Panel d illustrates the pocket formed by CC′ loop (dark blue) and FG loop (red) of CD22, with bound α2-6 siallylactose (sticks, green). The resdiues responsible for this non-canonical CC′ loop conformation is shown in sticks (yellow).

The examples of ICR complexes involving IgV domains described in the previous sections are protein-protein complexes. There are a few examples of immune receptors having IgV domains that recognize biologically important small molecules, suggesting the versatility of this domain to carve its front face to recognize molecules as big as proteins and small molecules of the size of an amino acid. The relatively flat canonical front face of the IgV/Ig like domain does not seem to support a cleft that can accommodate binding of small molecules. Hence, for the IgV domain to acquire such a property, it appears that a non-canonical conformational change is required to accommodate small molecules (Fig. 4a). This non-canonical conformation change was observed for the first time in the case of murine T-cell immunoglobulin and mucin domain containing protein-3 (mTIM-3), where unusual conformation of the CC′ loop creates a cleft for small molecule binding^63^. mTIM-3 is primarily expressed on Th1 and dendritic cells^64^. They bring about apoptosis in Th1 cells via downstream inhibitory signalling, when phosphatidylserine (PtdSer) expressed on cells destined for apoptosis, bind to mTIM-3^65^. As depicted in the Fig. 4a,b, the CC′ loop of mTIM-3 is peeled out of the IgV domain core towards FG loop of mTIM-3, forming a narrow pocket between CC′ loop and FG loop, which is known to bind PtdSer. As explained in the introduction, in a canonical IgV/Ig domain, the tip of CC′ loop and FG loop are separated approximately by 25 Å and these two loops are located at the two ends of the IgV domain (Figs. 1b, 4a). This binding pocket carved between the CC′ and FG loops is conserved in all TIM family proteins^63,66,67^. Note that such unusual peeling of C and C′ strands as well as the connecting loop away from the core would fundamentally require (1) change in the side-chain functionality of the core forming hydrophobic residues which are exposed to the solvent due to this non-canonical conformation (2) a strong interaction that can peel the CC′ loop away from its canonical position to form a pocket with FG loop. As expected, this unusual conformation of CC′ loop is stabilized by two non-canonical disulphide bonds between C_33_ of the C strand with C_44_ of the CC′ loop and C_91_ of F strand with C_39_ of the CC′ loop (PDB:3KAA) (Fig. 4b)^68^. These non-canonical disulphide bonds pull the CC′ loop from its canonical position and away from the core towards the FG loop. Further, few of the core-forming residues of the C and C′ strands and those from the core that interact with these residues in canonical IgV domains, are changed to hydrophilic residues. This pocket is further stabilized by few hydrogen bonds and salt-bridges. The CC′ loop of mTIM-3 comprising of _55_KGFCPWSQCTN_65_ (PDB:3KAA) contributes about 31.43% to the total mTIM-3 interaction-interface BSA and makes two potential hydrogen bonds with the serine moiety of PtdSer (Fig. 4b). W_41_, present on the tip of the CC′ loop acts as a gate for this pocket, regulating the entry of PtdSer. W_41_ in the closed conformation connects the CC′ loop and FG loop via hydrophobic interactions (PDB:2OYP)^63^. However, in the presence of PtdSer, W_41_ flips and rotates in such a manner that it moves away from the FG loop and allows PtdSer to enter the pocket (Fig. 4c). Mutagenesis studies suggested that mutation of _60_WSQ_62_ to homologous human sequence of VFE critically reduced the binding affinity of mTIM-3 to PtdSer^68^. These observations and experiments emphasize the role of this non-canonical CC′ loop in forming the pocket for an IgV domain to interact with a small molecule. It also plays a role of gating the entry of the small molecule into the pocket.

Another important example of CC′ loop of the IgV domain assuming a non-canonical conformation to form a small molecule binding cleft is that of CD22. CD22 is a receptor present on the B-cells and maintains a basal level of inhibition of B-cell activation. CD22 is a member of the sialic acid-binding immunoglobulin-like lectins family (Siglecs), found only on B-cells^69^ and binds specifically to α2-6 siallylactose. Since, CD22 is purported to bind to α2-6 siallylactose, a non-canonical feature is observed similar to that of mTIM-3, where the C, C′ strands and CC′ loop move away from the core to form a unique pocket (PDB:5VKM)^70^. The C and C′ strands of CD22 are bent towards the N-terminal forming a β-hairpin. This β-hairpin structure along with the FG loop forms a comparatively wide but shallow pocket, where α2-6 siallylactose binds to CD22 (Fig. 4d).

As mentioned earlier, the non-canonical conformation of the CC′ loop to form a pocket with FG loop could be due to the presence of hydrophobic residues like I_57_, F_59_, P_62_ and Y_64_ on the C strand and F_71_ on C′ strand in CD22 which forms a hydrophobic core on the front face (Fig. 4d). This hydrophobic core enables the C and C′ strand to move towards the FG loop. Furthermore, the presence of polar residues like N_61_, E_63_ and N_65_ on the C strand and D_72_ and T_74_ on the C′ strand, which would be facing the hydrophobic core of a canonical IgV domain, might have pushed the C and C′ strand away from the protein hydrophobic core due to steric clashes.

In a recent study that describes the structure of CD160:HVEM complex (PDB:6NG3)^71^, it was observed that a similar reorientation of C and C′ strands together with a single non-canonical disulphide bridge creates a pocket in the front-face of CD160 (Supplementary Fig. S2), which is wider than that is observed in mTIM-3 and CD22. The dimension of the HVEM being slightly smaller than the IgV domains, and obviously wider than the small molecules, requires complementary surface on CD160 that would exactly fit the dimensions of the HVEM. It is interesting to note that unlike TIM family members with two disulphide bridges on the front-face, how a relative wider surface is created using single disulphide bridge. Furthermore, point mutations on the CC′ loop of CD160, resulted in the reduced binding to HVEM. All these results suggest that nature uses the versatility of C and C′ strands together with CC′ loop to create ligand binding surface of different dimensions.

A question piqued our interest in these structures, why does the CC′ loop assume a non-canonical conformation to form a pocket? Why doesn’t the onus fall on FG loop to form this non-canonical conformation? Upon closer observation, it might be due to the presence of the canonical disulphide bond between the B strand and F strand of the IgV domain. This covalent bond might restrict the flexible movement of the FG loop and/or F strand. Hence, we propose that, since CC′ loop is not constrained by the presence of any covalent bonds or other rigidity conferring interactions, it is a prime candidate in the front face to assume non-canonical conformations to incorporate and provide additional affinity to its respective binding partner.

Along with this analysis, existing mutagenesis studies on various ICRs revealed that CC′ loop made key interactions with their binding partners (Supplementary Table 1). Here, of the 11 mutagenesis studies, nine of them revealed that mutations on CC′ loop resulted in either disruption of interaction or reduction in affinity. These mutations were selected purely based on their location at the receptor:ligand interface as observed in the complex structures without any importance to CC′ loop. It should also be noted that a mutagenesis study on CC′ loop, where CC′ loop of Nectin-2 was replaced with that of PVR showed increased affinity to TIGIT^45^, closely mimicking the higher affinity observed between TIGIT and PVR compared to TIGIT and Nectin-2. This result suggests that the rational modification of CC′ loop might help to produce ICRs with desired affinity.

### Modification of CC′ loop might lead to increase in binding affinity: A case study of TIGIT/Nectin pathway

**Figure 5:**
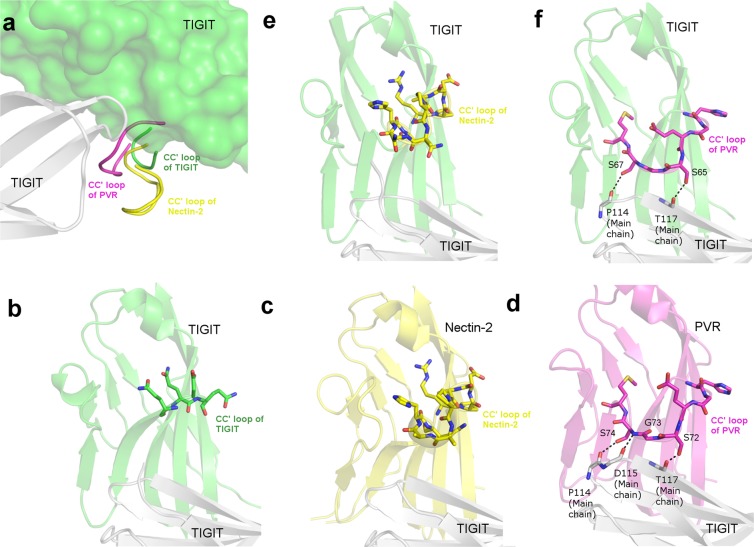
Modification of CC′ loop might lead to increase in binding affinity: Panel a illustrates the difference in the CC′ loops of TIGIT (ribbon, green), Nectin-2 (ribbon, yellow) and PVR (ribbon, magenta) when bound to TIGIT (cartoon, light grey). Since, all the three ligands have the same mode of interaction, TIGIT:Nectin-2 and TIGIT:PVR complexes were superposed on TIGIT(surface, green):TIGIT homodimer. Panel b, c and d illustrates the mode of interaction from experimentally determined structures of TIGIT (cartoon, green):TIGIT (cartoon, light grey), Nectin-2 (cartoon, yellow):TIGIT (cartoon, light grey) and PVR (cartoon, magenta):TIGIT (cartoon, light grey). The CC′ loops of the respective structures are shown in sticks. H-bond interactions of CC′ loop of PVR with TIGIT is indicated with black dotted line. Panel e and f depicts the best docked poses of T_N_:WT-T and T_P_:WT-T respectively where, modified TIGIT in both e and f panel is represented in green, cartoon and the spliced CC′ loops of Nectin-2 and PVR are represented in sticks, yellow and magenta respectively. Wild type TIGIT in both panels e and f is represented in light grey, cartoon. The interacting residues of T_P_ and TIGIT in panel F are represented in sticks.

T-cell immunoreceptor with Ig and ITIM domain (TIGIT) is an inhibitory receptor expressed on NK cells and certain subset of T-cells. TIGIT belongs to the PVR/Nectin family^72^ and is responsible for blocking the anti-tumor activity in NK cells^73^ and T-cells^72^. TIGIT binds to itself, PVR and Nectin-2 in a canonical mode where the Y/FP motif present on the FG loop of all the three proteins, recognizes a structurally conserved complementary cleft on the front face of their partners^38,45,46^. Superposition of human TIGIT:TIGIT, TIGIT:Nectin-2 and TIGIT:PVR structures, clearly suggests that TIGIT binds to all its binding partners and to itself in the same manner due to the ‘lock and key’ mode of interaction, but with differing binding affinity^38^. Among a few factors for these varied binding affinities between TIGIT and its ligands, the differing lengths and side-chain functionalities of the residues of CC′ loops of TIGIT, Nectin-2 and PVR (Fig. 5a) was proposed as a key factor^38,45^. This similarity in the mode of association of TIGIT with itself and with Nectin-2 and PVR gives us an opportunity to computationally investigate the impact of the CC′ loop on the binding affinity. Considering these observations, TIGIT/Nectin pathway presents itself as a prime example for investigating such a hypothesis. The CC′ loop of TIGIT is the shortest with 4 polar residues (_61_QQDQ_64_) (PDB:3RQ3), Nectin-2 has the longest CC′ loop with 9 residues (_72_RPDAPANHQ_80_) (PDB:5V52)^45^ and PVR has a 7-residue long CC′ loop (_69_HGESGSM_75_) (PDB:3UDW)^46^. The CC′ loop of TIGIT makes no contribution to the interaction-interface of the TIGIT homodimer, very weak Van der Waals interactions are provided by the residues of CC′ loop of Nectin-2 towards the interaction-interface of TIGIT:Nectin-2 heterodimer. However, a mix of Van der Waals and H-bond interactions are provided by the CC′ loop of PVR towards the interaction-interface of TIGIT:PVR heterodimer (Table 1) (Fig. 5b–d). This concept is well elucidated by the experimental swapping of CC′ loop of Nectin-2 with the CC′ loop of PVR. This particular Nectin-2 mutant displayed a higher affinity towards TIGIT than wild type Nectin-2^45^. In line with this mutagenesis study, we tried to apply a similar concept of CC′ loop swapping on TIGIT monomer. Since, TIGIT forms a weak homodimer and the contribution from its CC′ loop is low, could the binding affinity of TIGIT with itself be increased by splicing its CC′ loop with that of the CC′ loops of PVR and Nectin-2 on one of the protomers of the TIGIT homodimer? Experimentally determined structures of TIGIT:PVR (PDB:3UDW) and TIGIT:Nectin-2 (PDB:5V52) and TIGIT:TIGIT homodimer (PDB:3RQ3) were superposed in the crystallographic modelling program *Coot*. In two independent attempts, CC′ loops of one TIGIT monomer of the TIGIT homodimer (PDB:3RQ3) was swapped with the CC′ loop of Nectin-2 and PVR.

The complex structure thus created comprising of TIGIT with the CC′ loop of Nectin-2 (named T_N_) and wild type TIGIT (WT-T) was subjected to energy minimization using UCSF Chimera with Amber ff14SB forcefield. The same was done with the heterodimer containing TIGIT with the CC′ loop of PVR (T_P_) and WT-T. Since, the mode of binding of TIGIT:TIGIT homodimer and TIGIT:PVR/Nectin-2 heterodimers are similar, the resulting energy minimized structures of WT-T:WT-T, T_P_:WT-T and T_N_:WT-T were docked using RosettaDock via ROSIE server.

To examine the veracity of the docking algorithm, docking trials were also performed on experimentally determined TIGIT:TIGIT, TIGIT:Nectin-2 and TIGIT:PVR complexes. It was observed that the interface score was a more accurate parameter than the docking score, when the scores were compared with the docked poses which were in accordance (RMSD < 1) with the experimentally determined structures. Hence, these interface scores (more negative score, better the strength of interaction) were used as a reference in order to compare with that of the modified heterodimers (T_P_:WT-T and T_N_:WT-T). Results showed that the WT-T:WT-T had an interface score of −8.2. The best docked poses of T_N_:WT-T and T_P_:WT-T gave interface scores of −8.4 and −9.1 respectively (Fig. 5e,f respectively). It is interesting to note that difference in the interface scores of T_N_:WT-T and WT-T:WT-T (−0.2) is higher than that between T_P_:WT-T and WT-T:WT-T (−0.9), which indicates that the contribution of the CC′ loop of PVR plays an important role in the interaction-interface than the CC′ loops of TIGIT and Nectin-2. It can be expected that the differences in interaction due to other front face residues of Nectin-2 and PVR also contribute to the observed differences in affinity between TIGIT:Nectin-2 and TIGIT:PVR complexes. However, the docked T_N_:WT-T and T_P_:WT-T structures are similar to the experimentally determined (PDB:3UDW and 5V52 respectively) structures and maintain similar interactions seen between the CC′ loops of PVR and Nectin-2 with the WT-T. The interface scores are reflective of the fact that CC′ loop of Nectin-2 makes very few hydrophobic interactions, whereas CC′ loop of PVR makes significant amount of hydrophobic and hydrophilic interactions, which is also observed in the experimentally determined structures. This increase in the interaction score of T_P_:WT-T is due to the additional interactions made by the CC′ loop of PVR with the cognate TIGIT monomer, similar to what is seen in TIGIT:PVR. Conversely, it was observed that when the CC′ loop of PVR was replaced by the CC′ loop of TIGIT (P_T_) and docked with the WT-T in the canonical orientation, there were loss of important interactions which is also supported by interface score of −6.4. These interface scores indicate that the modifed versions of TIGIT (T_P_ and T_N_) have a higher binding affinity with its corresponding monomer of TIGIT (WT-T) than wild-type TIGIT itself. Interestingly, mere increase in the length of the CC′ loop doesn’t translate into increased binding affinity as the length of CC′ loop of PVR is shorter than that of Nectin-2. Functionalities of the residues present in the CC′ loop and their interactions with the cognate ligand appear to take precedence. These results might provide the motivation to proceed with wet lab experiments to confirm our hypothesis with not only TIGIT/Nectin pathway, but other medically relevant ICRs as well.

## Conclusion

The immune checkpoint blockade has brought about a paradigm shift in cancer therapy, however, rising reports of toxicity of mAbs coupled with its high cost, suspected resistance, requirement of high dose, poor solid tumour penetration and so on, have pushed for alternatives that can mimic the function of mAbs. Since, the soluble isoforms of these ICRs are naturally used by our immune system to tweak the immune response, rationally designed naturally occurring ICRs to mimic the function of therapeutic mAbs appears to be an effective approach. However, to design such a protein, sound structural information of the proteins and intuitive modification strategies are critical. In this study, the CC′ loop appears to influence the binding affinity of IgV domain containing ICRs in about half the ICR complexes analysed. Further, in about quarter of these complexes, the CC′ loop contributes more towards the interaction-interface than the FG loop. The CC′ loop is flexible enough to accommodate the incoming ligand and once bound, it appears to act like a clamp to increase the binding affinity of the complex. The CC′ loop does not arbitrarily move to accommodate the incoming ligand. Its movement is specific for a specific ligand, as clearly elucidated with the movement of CC′ loop of PVR, when bound to TIGIT (moves towards) and CD96 (moves away)^48^. In a few cases, the CC′ loop takes a preformed non-canonical conformation, as observed in the case of TIM-3 and CD22. This work attempts to provide novel insights for the rational design of soluble ICRs to mimic the role essayed by mAbs with an expected low toxicity and cost and suggests CC′ loop of the IgV domains of ICRs is the probable hot-spot for modification.

## Methodology

### Retrieving structures and complexes for the study

ICR complexes and other immune related receptors which contained at least one IgV domain were mined from literature^4^ and their apo structures and complexes were retrieved from worldwide protein databank (wwPDB)^74^. The presence of IgV domain in these retrieved structures were confirmed by InterPro-Protein sequence analysis and classification^75^. The secondary structure description as provided by PDB header was considered while determining the identity and length of the CC′ loop. The residue numbering in the PDB coordinate file was considered for the analysis and the same was used in the manuscript.

### Structure and interaction analysis

The interactions amongst the ICRs and their ligands were analysed using PDBe PISA^76^, where H-bond length not greater than 3.5 Å and not smaller than 2.5 Å were considered. Van der Waals interactions were considered if the two residues were separated by a distance of less than 5 Å. Coot^77^ and PyMol^78^ were used to visualize the complexes and their interactions. Buried surface area (BSA) of each monomer in the complex was considered for calculating the contribution of CC′ loop of the respective monomer which is indicative of Van der Waals interactions. The post-interaction movement of CC′ loop was considered significant, if the maximum deviation of the C_α_ atom of any residue which belongs to CC′ loop is more than 3 Å. The sequence logos highlighting the conservation of the residues of CC′ loop have been performed using WebLogo^79^.

### *In silico* modelling and energy minimization trials

*In silico* splicing of the CC′ loops of PVR and Nectin-2 onto one TIGIT monomer of the TIGIT homodimer was performed using Coot using experimentally solved structure of TIGIT homodimer in hexagonal space group (PDB: 3RQ3) as the template structure. The CC′ loops of PVR and Nectin-2 were excised and replaced the CC′ loop of TIGIT by superposing TIGIT homodimer structure with TIGIT:PVR (PDB:3UDW) and TIGIT:Nectin-2 (PDB:5V52) heterodimers independently. These *in silico* modified heterodimers of TIGIT (wild type TIGIT with TIGIT with CC′ loop of Nectin-2 or PVR) were subjected to energy minimization using UCSF Chimera’s Amber ff14SB forcefield^80^. These values were documented for further analysis.

### Protein-protein docking trials

As mentioned earlier, the mode of interaction of TIGIT homodimer and heterodimers are the same. Hence, the modified TIGIT heterodimers were docked using RosettaDock^81,82^ via ROSIE server^83^. Since, the modified heterodimers were derived from the exact pose of the experimentally determined structure (PDB:3RQ3), interface score was given paramount importance. The interface score was found to be more relevant when the docked poses were compared with experimentally determined structures. This parameter would help in inferring the trend associated with the impact of modification of the CC′ loop. The docked pose with the highest interface score which had a RMSD <1 with the submitted template structure, was considered for further investigations. Negative interface score indicates stronger interactions between the interfaces of the two proteins. Furthermore, thorough structural analysis was carried out on the selected docked pose to ascertain the number and type of interactions made by the CC′ loop with its binding partner. These interactions were compared with the interactions made by the CC′ loops of PVR and Nectin-2 with TIGIT as seen in experimentally determined structures (PDB:3UDW and 5V52 respectively) using PDBe PISA^76^ and PIC^84^.

## Supplementary information

Supplementary material

## Data Availability

UAR and SVK agree to make all protocols and data available for the readers.
