# Supplementary material for: The CC′ loop of IgV domains of the immune checkpoint receptors, plays a key role in receptor:ligand affinity modulation
Source: Sci Rep. 2019 Dec 16;9:19191. doi: 10.1038/s41598-019-54623-y (PMC6914781; doi:10.1038/s41598-019-54623-y)
Supplement: Supplementary file 1 — Supplementary material [file 41598_2019_54623_MOESM1_ESM.docx]

**Supplementary material**

**The CC′ loop of IgV domains of the immune checkpoint receptors, plays a key role in receptor:ligand affinity modulation.**

**Shankar V. Kundapura^1,2^ and Udupi A. Ramagopal^1 *^**

^1^Division of Biological Sciences, Poornaprajna Institute of Scientific Research, #4, 16^th^ Cross, Sadashivnagar, Bangalore 560080, India

^2^Manipal Academy of Higher Education, Manipal, Karnataka, India-576104

**Architecture of IgV domains**:

Nature has utilized this Ig fold to give rise to proteins with diverse functions. This makes IgSF one of the largest family of proteins, with about 2% genes of the human genome belonging to IgSF^1^. The IgSF is broadly classified into four structural sets namely, IgC1, IgC2, IgV and IgI^2,3^. They are differentiated based on the number of β-strands and their association and the linear residue distance between the conserved cysteines which form the canonical disulphide linkage. The Ig fold is a twisted β sandwich of roughly 100-130 amino acids, comprising of two β sheets made up of seven β-strands named A to G where, A, B and E strands form one β sheet and G, F and C strands form the other β sheet (Figure S1a-c). A canonical disulphide linkage observed between B and F strands, connects the two sheets. In almost all the cases including antibodies, IgV is the functional domain and other domains like IgC1 and IgC2 are used as the structural support. There are two additional β-strands namely, C′ and C′′ present in the IgV domain (Figure S1b), which makes the front face wider, comprising of A′, G, F, C, C′ and C′′ strands of an IgV domain (Figure S1d)^4^. This domain has been found in many protein families like ICRs, antibodies, nanobodies, cell adhesion molecules and cell surface receptors^5^.


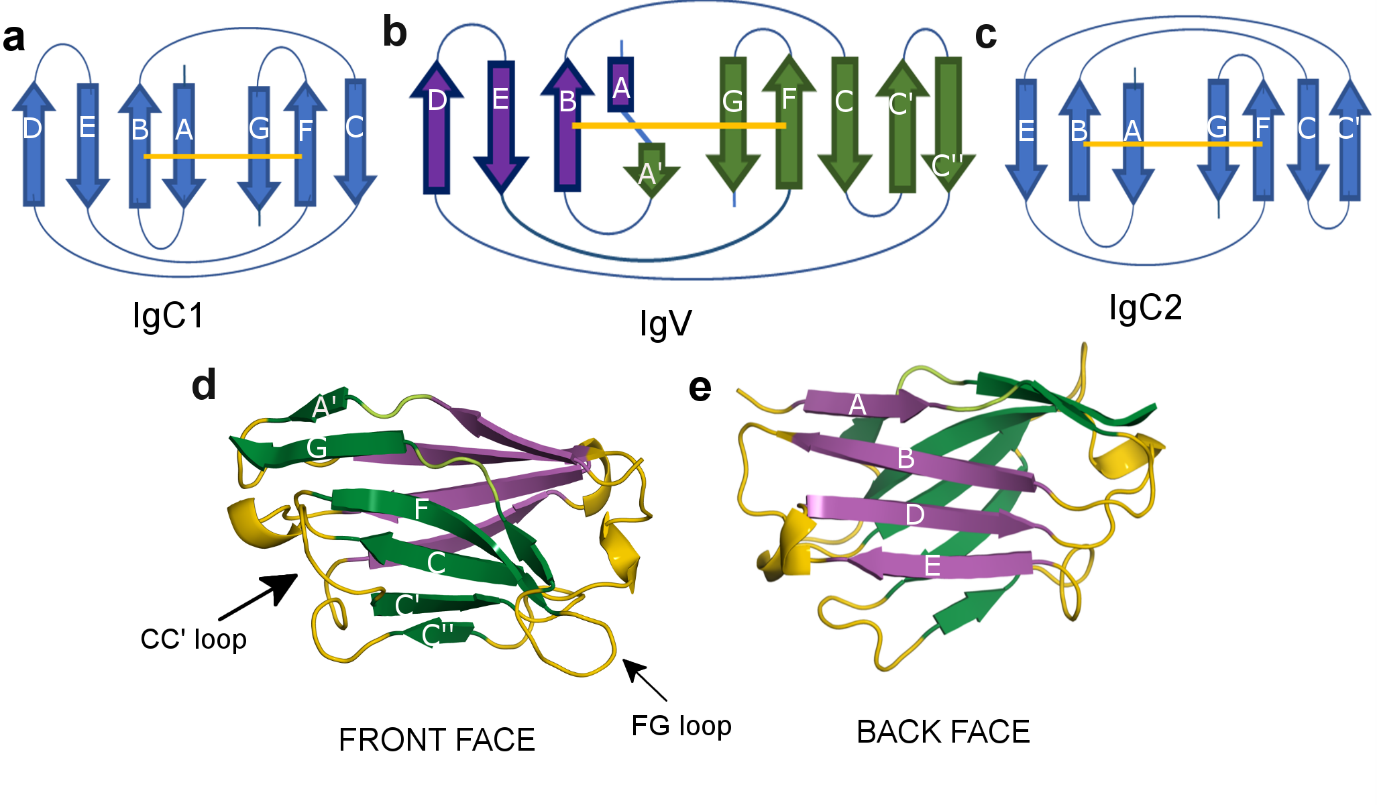
Supplementary figure S1: Organisation of IgV domain: Panels a, b and c illustrate the secondary structure organisation of IgC1 IgV and IgC2 domains respectively. The yellow line represents the disulphide linkage from strand B to strand F in the panels a, b and c. Panel d denotes the front face (Green, ribbon) and panel E illustrates the back face (Purple, ribbon) of IgV domain. Note that the front face is flanked by the FG and CC′ loops and are located at the opposite ends of the front face (Panel d).


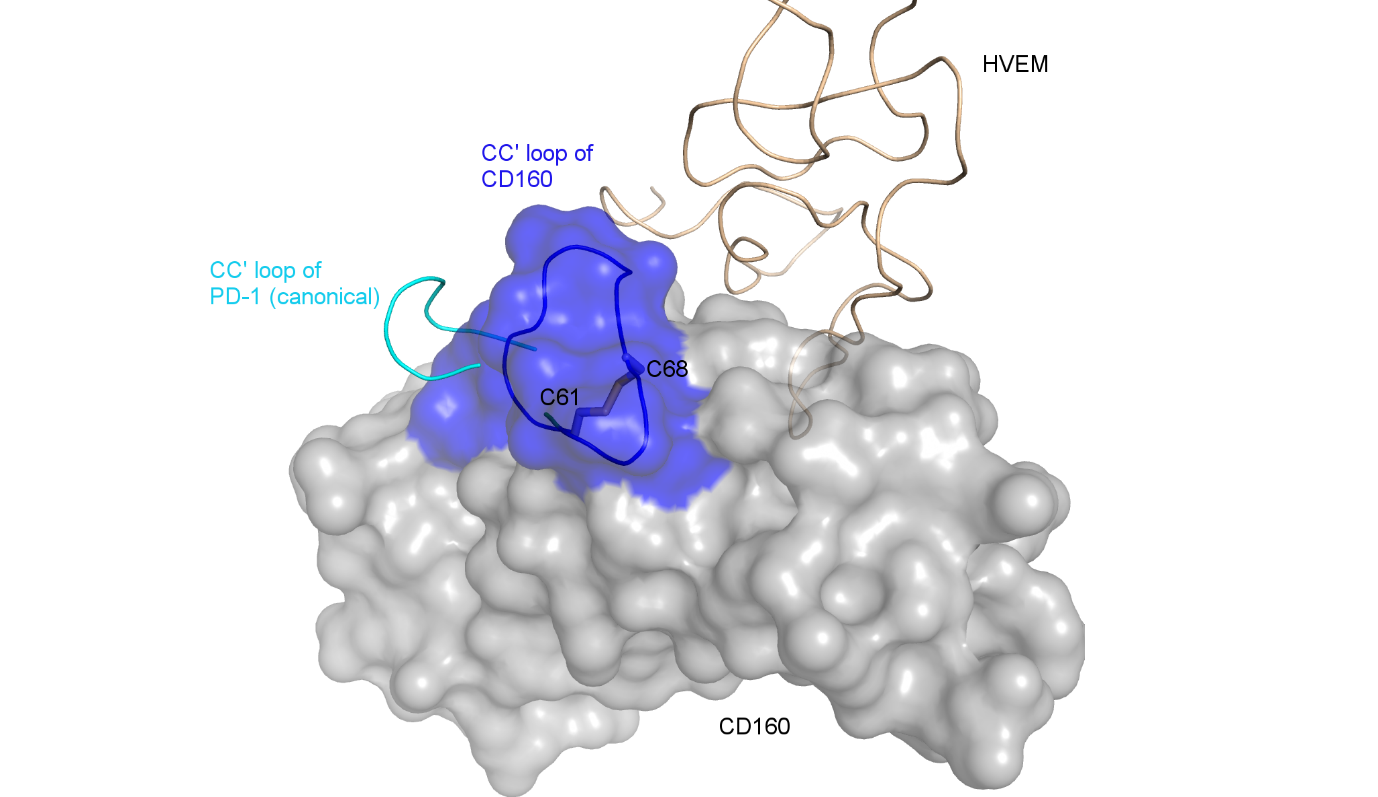


Supplementary figure S2: Complex of CD160:HVEM: CD160 (grey, surface) is complexed with HVEM (wheat, ribbon), where the CC′ loop is highlighted in blue and is held in a non-canonical conformation by two cysteines, C61 and C68 (blue, stick). PD-1 (PDB: 3RRQ) was superposed onto CD160 to denote the canonical CC′ loop (cyan, ribbon). Unlike mTIM-3 and CD22, the CC′ loop of CD160 is propped up using one disulphide linkage.

Table 1:Compilation of existing mutagenesis studies on CC′ loop of various IgV containing ICRs

| Sl. No. | Protein | Mutagenesis of CC′ loop | Effect of mutation |
| --- | --- | --- | --- |
| 1. | mTIM-3 | _60_WSQ_62_ to VFE | Reduces binding to PtdSer^6^ |
| 2. | hCD160 | D_63_A,  D_67_A,  D_67_R | Significantly reduces binding to hHVEM^7^ |
| 3. | rCD2 | E_41_R,  K_43_E | No binding to rCD48^8^ |
| 4. | hCD2 | K_43_A | Partial loss of binding to hCD58^9^ |
| 5. | hNectin-2 | CC′ loop swapped with that of PVR | Increases binding to hTIGIT^10^ |
| 6. | m2B4 | R_43_A | No change in binding to mCD48^11^ |
| 7. | hCD58 | K_32_A | Partial loss of binding to hCD2^9^ |
| 8. | rCD48 | K_41_E,  K_41_R | No binding to rCD2^8^ |
| 9. | hNTB-A | E_37_A | Significantly reduced Homodimerization^12^ |
| 10. | hCRTAM | F_39_A | Significantly reduced binding to hNecl-2^13^ |
| 11 | mCD226 | H_68_A | Significantly reduces binding to hPVR^14^ |

References

1. Beck, S. Immunogenomics: towards a digital immune system. *Novartis Found. Symp.* **254**, 223–30; discussion 230-3, 250–2 (2003).

2. Harpaz, Y. & Chothia, C. Many of the Immunoglobulin Superfamily Domains in Cell Adhesion Molecules and Surface Receptors Belong to a New Structural Set Which is close to That Containing Variable Domains. *J. Mol. Biol.* **238**, 528–539 (1994).

3. Bateman, A., Eddy, S. R. & Chothia, C. Members of the immunoglobulin superfamily in bacteria. *Protein Sci.* **5**, 1939–41 (1996).

4. Natarajan, K., Mage, M. G. & Margulies, D. H. Immunoglobulin Superfamily. in *eLS* 1–7 (John Wiley & Sons, Ltd, 2015). doi:10.1002/9780470015902.a0000926.pub2

5. Teichmann, S. A. & Chothia, C. Immunoglobulin Superfamily Proteins in Caenorhabditis elegans. *J. Mol. Biol.* **296**, 1367–1387 (2000).

6. DeKruyff, R. H. *et al.* T Cell/Transmembrane, Ig, and Mucin-3 Allelic Variants Differentially Recognize Phosphatidylserine and Mediate Phagocytosis of Apoptotic Cells. *J. Immunol.* **184**, 1918–1930 (2010).

7. Liu, W. *et al.* Structural Basis of CD160:HVEM Recognition. *Structure* **27**, 1–10 (2019).

8. Anton van der Merwe, P., McNamee, P. N., Davies, E. A., Barclay, A. N. & Davis, S. J. Topology of the CD2–CD48 cell-adhesion molecule complex: implications for antigen recognition by T cells. *Curr. Biol.* **5**, 74–84 (1995).

9. Wang, J. H. *et al.* Structure of a heterophilic adhesion complex between the human CD2 and CD58 (LFA-3) counterreceptors. *Cell* **97**, 791–803 (1999).

10. Deuss, F. A., Gully, B. S., Rossjohn, J. & Berry, R. Recognition of nectin-2 by the natural killer cell receptor T cell immunoglobulin and ITIM domain (TIGIT). *J. Biol. Chem.* **292**, 11413–11422 (2017).

11. Velikovsky, C. A. *et al.* Structure of Natural Killer Receptor 2B4 Bound to CD48 Reveals Basis for Heterophilic Recognition in Signaling Lymphocyte Activation Molecule Family. *Immunity* **27**, 572–584 (2007).

12. Cao, E. *et al.* NTB-A Receptor Crystal Structure: Insights into Homophilic Interactions in the Signaling Lymphocytic Activation Molecule Receptor Family. *Immunity* **25**, 559–570 (2006).

13. Zhang, S. *et al.* Competition of cell adhesion and immune recognition: Insights into the interaction between CRTAM and nectin-like 2. *Structure* **21**, 1430–1439 (2013).

14. Wang, H. *et al.* Binding mode of the side-by-side two-IgV molecule CD226/DNAM-1 to its ligand CD155/Necl-5. *Proc. Natl. Acad. Sci. U. S. A.* **116**, 988–996 (2019).
